# Structural analyses of adenylate kinases from Antarctic and tropical fishes for understanding cold adaptation of enzymes

**DOI:** 10.1038/s41598-017-16266-9

**Published:** 2017-11-22

**Authors:** Sojin Moon, Junhyung Kim, Euiyoung Bae

**Affiliations:** 1 Department of Agricultural Biotechnology, Seoul National University, Seoul, 08826 Korea; 2 Research Institute of Agriculture and Life Sciences, Seoul National University, Seoul, 08826 Korea; 3 Present Address: iNtRON Biotechnology, Inc., Seongnam, 13202 Korea

## Abstract

Psychrophiles are extremophilic organisms capable of thriving in cold environments. Proteins from these cold-adapted organisms can remain physiologically functional at low temperatures, but are structurally unstable even at moderate temperatures. Here, we report the crystal structure of adenylate kinase (AK) from the Antarctic fish *Notothenia coriiceps*, and identify the structural basis of cold adaptation by comparison with homologues from tropical fishes including *Danio rerio*. The structure of *N*. *coriiceps* AK (AKNc) revealed suboptimal hydrophobic packing around three Val residues in its central CORE domain, which are replaced with Ile residues in *D*. *rerio* AK (AKDr). The Val-to-Ile mutations that improve hydrophobic CORE packing in AKNc increased stability at high temperatures but decreased activity at low temperatures, suggesting that the suboptimal hydrophobic CORE packing is important for cold adaptation. Such linkage between stability and activity was also observed in AKDr. Ile-to-Val mutations that destabilized the tropical AK resulted in increased activity at low temperatures. Our results provide the structural basis of cold adaptation of a psychrophilic enzyme from a multicellular, eukaryotic organism, and highlight the similarities and differences in the structural adjustment of vertebrate and bacterial psychrophilic AKs during cold adaptation.

## Introduction

Extremophiles are organisms that can thrive in extreme environmental conditions of various physical and chemical parameters such as temperature, pressure, pH, and salinity^1–3^. Among the extremophiles, psychrophiles and thermophiles, which are tolerant of low and high temperatures, respectively, have been of particular interest as they have potential in biotechnological applications^2–5^. Proteins isolated from psychrophilic and thermophilic organisms can remain physiologically functional at extreme temperatures, which are detrimental to mesophilic proteins from organisms living at moderate temperatures^6–9^. Hence, psychrophilic and thermophilic proteins are desirable for use in many academic and industrial settings that require biological activity at the extreme temperatures^8,10–12^.

To evaluate the molecular basis for the temperature adaptation of psychrophilic and thermophilic proteins, numerous comparative studies have performed comparisons with their mesophilic homologues^13–18^. Modifications of intramolecular interactions such as hydrogen bonds, electrostatic interactions, and hydrophobic contacts have been identified as the main structural mechanisms of cold and heat adaptation, but the various individual structural features change unpredictably to different extents in different proteins^13–18^. The alterations of intramolecular interactions are the results of structural adjustments that allow for appropriate flexibility to ensure physiological function at different temperatures^6–8,18–21^. Psychrophilic proteins tend to exhibit fewer intramolecular interactions than their mesophilic homologues, which, at low temperatures, would become too inflexible to perform the dynamic movements required for their biological functions^8,11,16–18,21,22^.

Previously, we reported the crystal structures, thermal denaturation midpoints (T_m_’s), temperature-activity profiles, and molecular dynamics trajectories of homologous adenylate kinases (AKs) from psychrophilic, mesophilic, and thermophilic bacteria^23–26^. The thermal stabilities and temperature optima for catalytic activities of the enzymes scaled with the environmental temperatures of their source organisms^23,25,26^. The psychrophilic AK revealed the lowest apolar buried surface area of the trio^23^, suggesting that a reduction in the number of hydrophobic contacts played a role in cold adaptation, which is generally defined as high catalytic activity at low temperatures^8,11,16,17^. However, the residue substitution(s) responsible for its decreased thermal stability have not been identified.

AK is a small enzyme that catalyzes a reversible phosphoryl transfer reaction between ATP/AMP and two ADPs. The tertiary structure of AK can be subdivided into three domains: CORE, AMP_bind_, and LID^27^. The static CORE domain provides substrate-binding sites, and the dynamic AMP_bind_ and LID domains close over the AMP and ATP sites, respectively, upon substrate binding^28–30^. AK is ubiquitous in all three kingdoms of life. Bacterial AKs are monomeric with a long LID domain^31^, whereas archaeal AKs form trimers containing a short LID domain^32^. Vertebrates have several AK isoforms. AK1 is the most abundant cytosolic AK isozyme, exists in a monomeric state, and contains a short LID domain^33,34^.

In this study, we solved the crystal structure of AK1 from the Antarctic fish *Notothenia coriiceps* (AKNc)^35^, and characterized its thermal stability and temperature–activity profile. To identify the structural adjustments important for its cold adaptation, AKNc was compared with homologous AKs from the following tropical fishes: *Poecilia reticulata* (AKPr)^36^, *Xiphophorus maculatus* (AKXm)^37^, and *Danio rerio* (AKDr)^38^, whose crystal structure was also determined in the present study. The habitat temperature of the Antarctic *N*. *coriiceps* (−1.5 to +1 °C)^39^ is significantly lower than typical living temperatures of the tropical species (>15 °C)^40–42^. Our results also highlight the similarities and differences in the structural changes related to temperature adaptation in vertebrate and bacterial AKs, indicating that the reduced thermal stability of psychrophilic enzymes may or may not be an adaptive trait depending on structural mechanism of catalytic activity.

## Results

### AKs from Antarctic and tropical fishes exhibited high sequence similarities but disparate thermal stabilities

**Figure 1:**
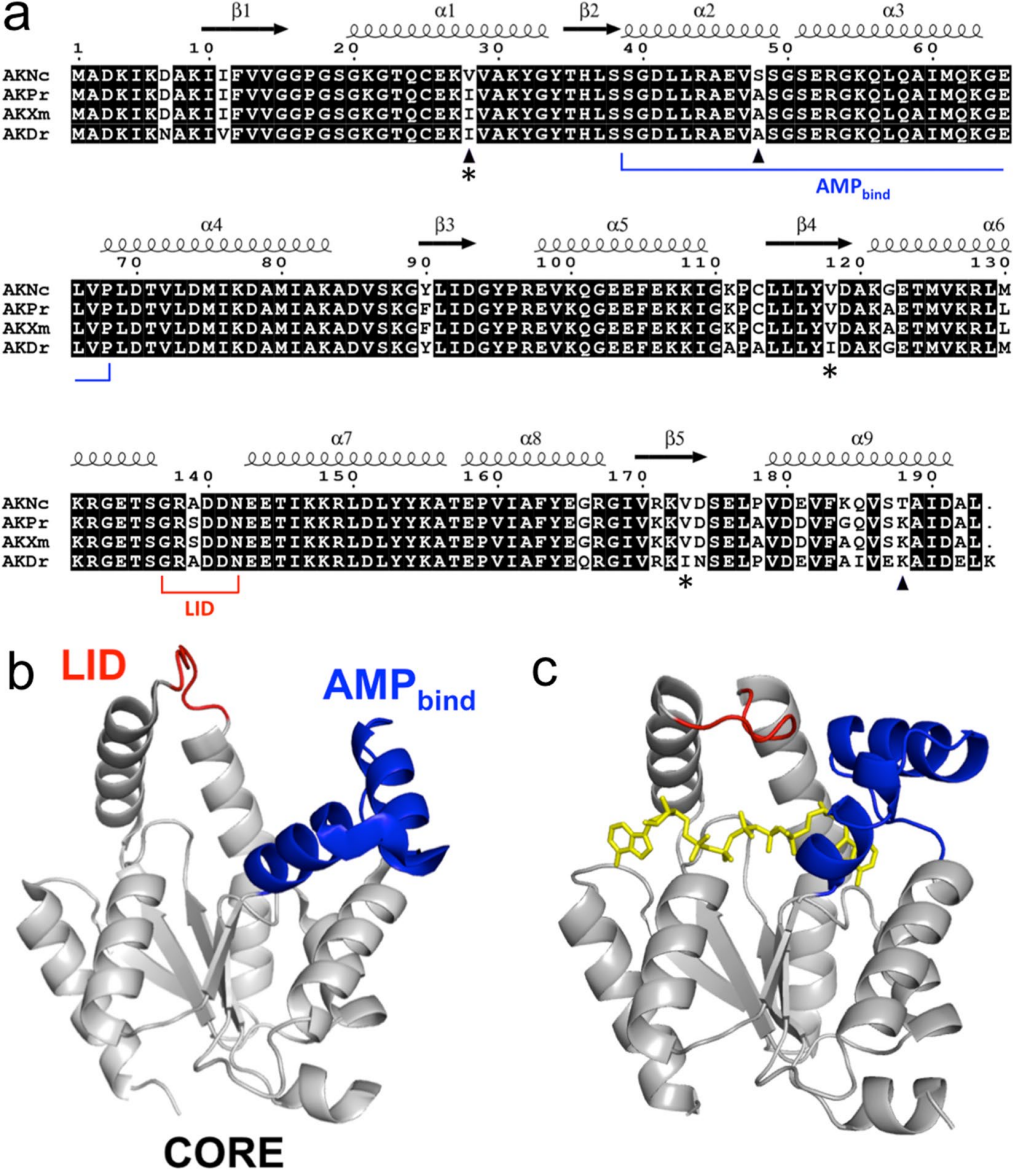
Sequence and dynamics of AKs. (**a**) Sequence alignment of AKNc from the Antarctic fish *N*. *coriiceps* and its homologues from tropical fishes including AKDr. Secondary structural elements are indicated based on AKNc. Three positions (residues 28, 48, and 188) at which the amino acid is conserved in the three tropical AKs but not in AKNc are indicated by triangles. The three Val residues (Val28, Val118, and Val173) around which hydrophobic packing is not optimal in the AKNc are indicated by asterisks. Residues of the AMP_bind_ domain (residues 39–68) and the LID (residues 137–142) are also indicated. The other residues belong to the CORE domain (residues 1–38, 69–136, and 143–193). (**b**,**c**) Structural dynamics of AK during a catalytic cycle. Open (**b**) and closed (**c**) conformations of AK are depicted using the crystal structures of porcine (Protein Data Bank code 3ADK) and human (Protein Data Bank code 1Z83) AK1 homologues, respectively. The CORE, AMP_bind_, and LID domains are shown in grey, blue, and red, respectively. Ap_5_A molecule bound to the active site is shown in yellow.

The amino acid sequence of AKNc has been aligned with those of the three homologous AKs from tropical fishes in Fig. 1. The sequences of the Antarctic and tropical AKs exhibit high-level similarity. The sequence identity between AKNc and AKDr is 92%, and AKNc shares 94% sequence identities with the other two tropical AK homologues, AKXm and AKPr. These two tropical AKs are identical except at only one position (residue 184) and, interestingly, share lower sequence identities (90%) with the tropical AKDr than with the Antarctic AKNc. Notably, the N- and C-terminal regions are the most variable in the sequence alignment of the AKs. AKNc and AKDr differ by 15 residues, more than two thirds of which are located within 30 residues from the N- and C-termini of the amino acid sequence. Only 11 residues differ between AKNc and the other two tropical AKs, and six of them are found in the N- and C-terminal regions.

The T_m_ values of the AKs were measured by circular dichroism (CD) spectroscopy (Fig. S1). The T_m_ of AKNc was significantly lower than those of homologues from tropical fishes (Table 1), indicating the thermal stability of AKNc is substantially reduced compared with those of the other three AKs. Among the tropical AKs, AKDr was the most thermally stable. The difference in T_m_ between AKDr and AKNc was 11.3 °C. The T_m_ values of AKXm and AKPr were 9.2 °C and 7.0 °C, respectively, higher than that of AKNc. In a previous study of bacterial AKs, the T_m_ difference was only 4.3 °C between psychrophilic and mesophilic homologues^23^. These results suggest that the thermal stabilities of the fish AKs reflect the temperature preferences of their source organisms, as the thermal transition of the Antarctic AKNc occurred at a substantially lower temperature than those of its homologues from tropical fishes.

**Table 1:** T_m_ values of WT and mutant AKs from the Antarctic and tropical fishes.

AK	Mutation(s)	T_m_ (°C)	ΔT_m_ (°C)^a^
AKNc	WT	33.8	0.0
	V28I	38.8	5.0
	S48A	36.0	2.2
	V118I	36.6	2.8
	V173I	36.2	2.4
	T188K	30.7	−3.1
	V118I/V173I	41.4	7.6
	V28I/V118I/V173I	45.1	11.3
AKPr	WT	40.8	7.0
AKXm	WT	43.0	9.2
AKDr	WT	45.1	11.3
	I28V/I118V/I173V	36.2	2.4

^a^Difference from the T_m_ of WT AKNc.

### Structural analyses revealed suboptimal hydrophobic packing in the central CORE domain of AKNc

To assess the structural basis of cold adaptation, we determined the crystal structures of AKNc and AKDr to resolutions of 1.99 and 1.75 Å, respectively, using molecular replacement. Data collection and refinement statistics are summarized in Table 2. The asymmetric units of both structures contain two independent AK monomers. The root mean square deviation (RMSD) values of the Cα atomic positions between the two monomers were only ~0.6 Å for both AKNc and AKDr, indicating that the two monomeric structures in the same asymmetric units are very similar. We hereafter describe only those (chain A’s), which exhibit lower average B factors.

**Table 2:** Data collection and refinement statistics^a^.

	AKNc	AKDr	AKNc V28I/V118I/V173I
Space group	P4_1_2_1_2	C2	P4_1_2_1_2
Unit cell parameters (Å)	a = b = 105.5, c = 84.3	a = 100.6, b = 52.5, c = 89.3,	a = b = 105.4, c = 83.8
Wavelength (Å)	1.0000	0.9793	0.9793
**Data collection statistics**			
Resolution range (Å)	50.00-1.99 (2.06-1.99)	50.00-1.75 (1.81-1.75)	50.00-1.90 (1.97-1.90)
Number of reflections	33142 (3227)	41960 (4159)	37833 (3703)
Completeness (%)	99.9 (100.0)	99.8 (99.7)	99.9 (100.0)
R_merge_^b^	0.078 (0.491)	0.115 (0.756)	0.090 (0.781)
Redundancy	14.1 (12.8)	7.1 (7.0)	7.1 (7.1)
Mean I/σ	49.4 (7.5)	19.6 (3.8)	24.9 (3.5)
**Refinement statistics**			
Resolution range (Å)	50.00-1.99	50.00-1.75	50.00-1.90
R_cryst_^c^/R_free_^d^ (%)	17.1/20.8	18.6/22.9	17.5/21.1
RMSD bonds (Å)	0.022	0.020	0.022
RMSD angles (deg)	2.4	2.1	2.4
Average B factor (Å^2^)	33.6	31.3	27.8
Number of water molecules	173	108	129
Ramachandran favored (%)	98.6	97.6	98.1
Ramachandran allowed (%)	1.4	2.4	1.9

^a^Values in parentheses are for the highest-resolution shell.

^b^R_merge_ = ∑_h_∑|I_i_(h) − < I(h) >|/∑_h_∑_i_I_i_(h), where I_i_(h) is the intensity of an individual measurement of the reflection and < I(h) > is the mean intensity of the reflection.

^c^R_cryst_ = ∑_h_||F_obs_| − |F_calc_||/∑_h_|F_obs_|, where F_obs_ and F_calc_ are the observed and calculated structure factor amplitudes, respectively.

^d^R_free_ was calculated as R_cryst_ using 5% of the randomly selected unique reflections that were omitted from structure refinement.

**Figure 2:**
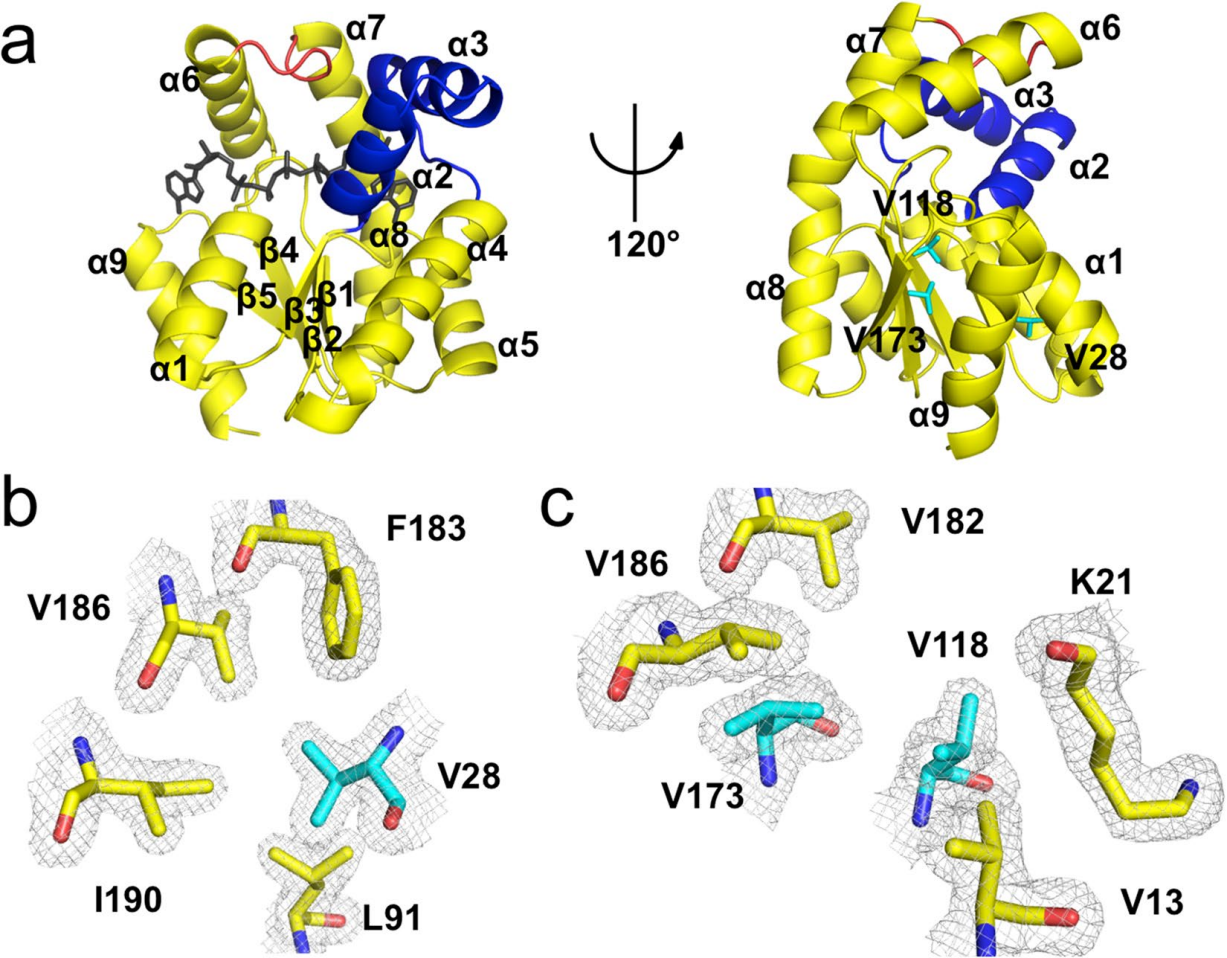
Crystal structure of the Antarctic AKNc. (**a**) Overall structure of AKNc. The CORE (residues 1–38, 69–136, and 143–193), AMP_bind_ (residues 39–68) and LID (residues 137–142) domains are shown in yellow, blue, and red, respectively. The co-crystallized Ap_5_A molecule bound to the active site is shown in black (left), but not in the 120°-rotated model (right), to reveal the AK structure more clearly. The three Val residues for which hydrophobic contacts are suboptimal are highlighted in cyan in stick representations (right). (**b**,**c**) Close-up views of the hydrophobic environment around Val28 (**b**) and Val118/Val173 (**c**). The Val residues interact hydrophobically with other residues in the CORE domain, but there is room for improvement in hydrophobic packing. The 2mF_obs_ − DF_calc_ map is contoured at 1.0 σ.

**Figure 3:**
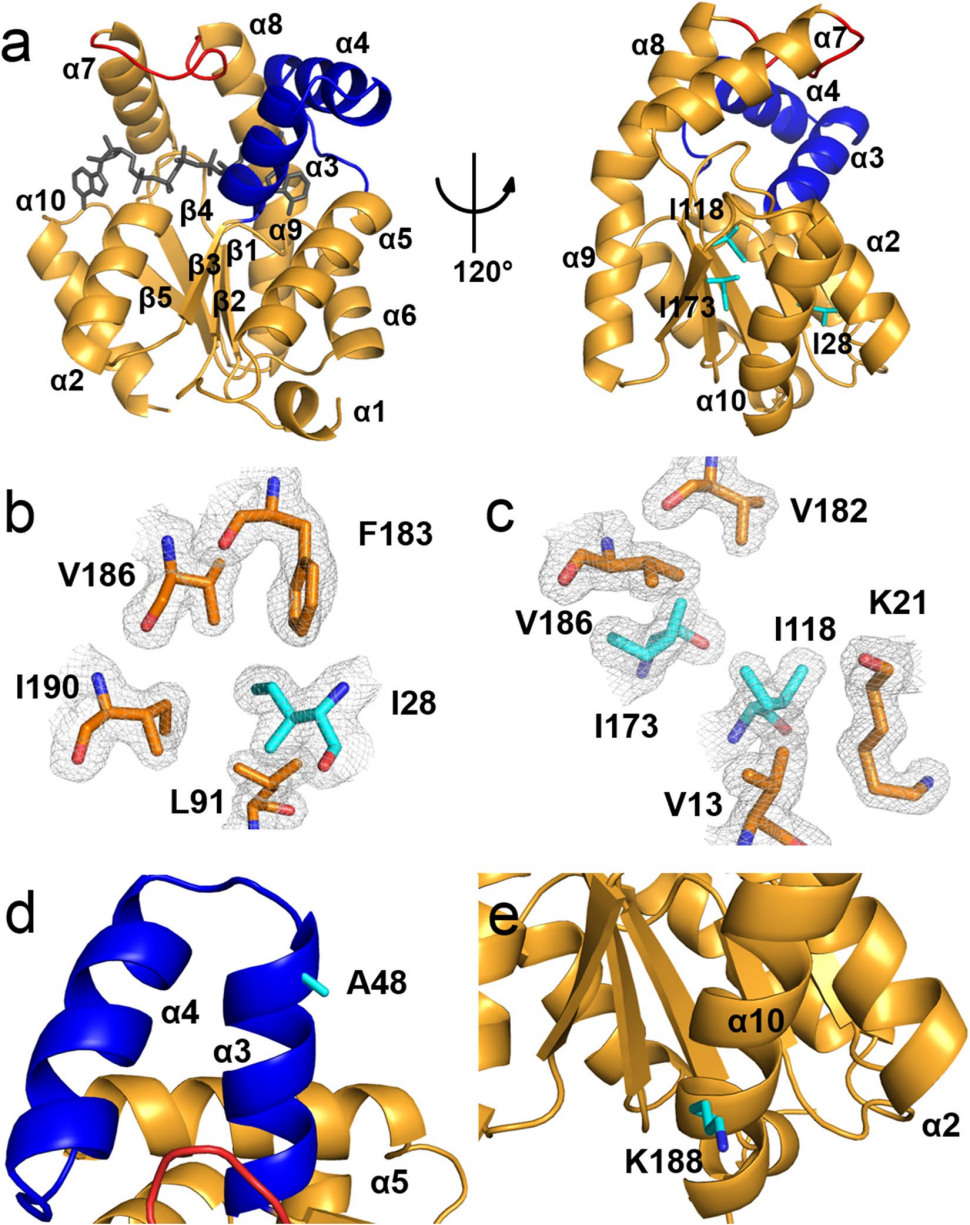
Crystal structure of the tropical AKDr. (**a**) Overall structure of AKDr. The CORE (residues 1–38, 69–136, and 143–194), AMP_bind_ (residues 39–68) and LID (residues 137–142) domains are shown in orange, blue, and red, respectively. The co-crystallized Ap_5_A molecule is shown in black. (**b**,**c**) Hydrophobic contacts of Ile28 (**b**) and Ile118/Ile173 (**c**) in the CORE domain of the AKDr structure. The three Ile residues are substituted to Val residues in AKNc. The 2mF_obs_−DF_calc_ map is contoured at 1.0 σ. (**d**,**e**) Close-up views of Ala48 (**d**) and Lys188 (**e**) in the crystal structure of AKDr. Ala48 and Lys188 are conserved among the three tropical AKs including AKDr, but not in AKNc. The side chains of Ala48 and Lys188 are exposed to the solvent, and do not interact closely with residues that are located distantly in the amino acid sequence.

The chain folds of AKNc and AKDr are essentially identical to those of its homologues (Figs 2a, 3a and S2). The structures comprised the characteristic three-domain arrangement: the CORE (residues 1–38, 69–136, and 143–193), AMP_bind_ (residues 39–68), and LID (residues 137–142) domains. The CORE domain consists of a five-stranded parallel β-sheet (β1–5) and seven α-helices (α1, α4–9), and the AMP_bind_ domain includes two α-helices (α2, α3). The LID domain is a short loop connecting α6 and α7 helices, as is true of other AK1 structures. The co-crystallized ligand P^1^,P^5^-di(adenosine 5′)-pentaphosphate (Ap_5_A), which mimics both AMP and ATP substrates, is bound to the active site and covered by the AMP_bind_ and LID domains, indicating that the crystal structures of AKNc and AKDr adopt the closed conformational state of AK^28^.

To identify key structural features for the cold adaptation of AKNc, we focused on amino acid residues conserved among the three tropical AKs, but not in the Antarctic AKNc. We found three such positions (residues 28, 48, and 188) in the sequence alignment (Fig. 1a). The three AKs from the tropical fishes including AKDr have Ile28, Ala48, and Lys188 at these positions, whereas AKNc has Val28, Ser48, and Thr188. We thought that these residue substitutions might be related to the temperature adaptation of the AKs. In the structure of AKNc, Val28 in the N-terminal α1 helix interacts closely (<4 Å) with conserved hydrophobic residues in the β3 strand (Leu91) and the C-terminal α9 helix (Phe183, Val186, and Ile190) (Fig. 2b). Moreover, the Val-to-Ile mutation at this position (residue 28) found in the tropical homologues is expected to improve the hydrophobic packing in the CORE domain. In the structure of AKDr, the longer side chain of Ile28 enhances the hydrophobic contacts with the residues in β3 and α9, and interacts more closely with hydrophobic residues in β1 and β4 (Fig. 3b). In contrast, Ala48 and Lys188 are largely exposed to the solvent in the AKDr structure, and do not make close contacts with residues that are located distantly in the polypeptide (Fig. 3d,e), indicating that mutations at these two positions in AKNc may not exert significant effects on the intramolecular interactions.

In the CORE domain of AKNc, we found two additional Val residues (Val118 and Val173), around which hydrophobic packing could be improved by mutations (Fig. 2c). At these positions, AKXm and AKPr also have Val residues, but AKDr, which is the most thermally stable of the three tropical AKs, has Ile residues. Val118 and Val173 of AKNc are adjacent to each other (<4 Å) in β4 and β5, respectively. Their side chains are found in the hydrophobic interior of the CORE domain, making contacts with residues in β1, α1, and α9 such as Val13, Lys21, Val182, and Val186. However, there is room for improvement in hydrophobic packing around the two Val residues (Val118 and Val173). Val-to-Ile mutations at these positions would most likely result in closer hydrophobic contacts with residues in the β strands (β1, β4, β5) and the two terminal helices (α1, α9). In the crystal structure of AKDr, Ile118 and Ile173 improve CORE packing cooperatively by increasing hydrophobic interactions between them as well as with other residues (Fig. 3c). Taken together, the structural analyses of AKNc and AKDr suggest that hydrophobic CORE packing is not optimal in AKNc, and is important in thermal stability of fish AKs.

### Improvement in hydrophobic CORE packing increased thermal stability of AKNc

To investigate the role of hydrophobic packing around the three Val residues in temperature adaptation, we generated a series of AKNc mutants, in which the Val residues were substituted to Ile residues individually or collectively, and measured their T_m_ values by CD spectroscopy (Table 1 and Fig. S1). The V28I mutation increased the thermal stability of AKNc considerably, as indicated by a 5.0 °C increase in T_m_ compared with that in the wild-type (WT). The enhancement of thermal stability resulting from the other two individual Val-to-Ile mutations was relatively modest. The V118I and V173I mutations increased the T_m_ of AKNc by 2.8 °C and 2.4 °C, respectively. However, the AKNc mutant with both the V118I and the V173I mutations exhibited an increase in T_m_ of 7.6 °C relative to the WT AKNc. This value is greater than the sum of T_m_ increases conferred by the V118I and V173I mutations individually, indicating a synergistic effect of the two mutations on the overall thermal stability. This is consistent with the structural analyses since the two residues are located close to each other (<4 Å) in the crystal structures (Fig. 2c).

The three Val-to-Ile mutations in combination resulted in the greatest thermal stability of AKNc (Table 1 and Fig. S1). The T_m_ value of the V28I/V118I/V173I mutant was 11.3 °C higher than that of the WT AKNc, and was identical to that of AKDr, the most thermally stable homologue of the three tropical AKs. This observation suggests that the suboptimal hydrophobic packing around the three Val residues in the CORE domain is important for the reduced thermal stability of AKNc compared with that of its homologues from tropical fishes. We also tested the role of the hydrophobic CORE packing in thermal stability in the opposite direction. We produced an AKDr mutant in which Ile28, Ile118 and Ile173 residues were replaced with Val residues, and determined its T_m_ value (Table 1 and Fig. S1). The reverse triple mutation significantly reduced the thermal stability of AKDr, as indicated by a decrease in T_m_ of 8.9 °C relative to the WT enzyme, confirming the importance of the CORE packing in the overall stability of the fish AKs.

We also measured the thermal stabilities of S48A and T188K mutants of AKNc to test the effect of residue substitution at these positions. The three tropical AKs have Ala48 and Lys188, but AKNc has Ser48 and Thr188. The T_m_ value of the S48A mutant was 2.2 °C higher than that of the WT AKNc, and the T188K mutation decreased the thermal stability of AKNc by 3.1 °C (Table 1). These results support the hypothesis that residues at these two positions may not be critical for overall thermal stability as they are not involved in intramolecular interactions connecting distant regions of the polypeptide, and suggest that the three Val residues play more important roles in the cold adaptation of AKNc.

**Figure 4:**
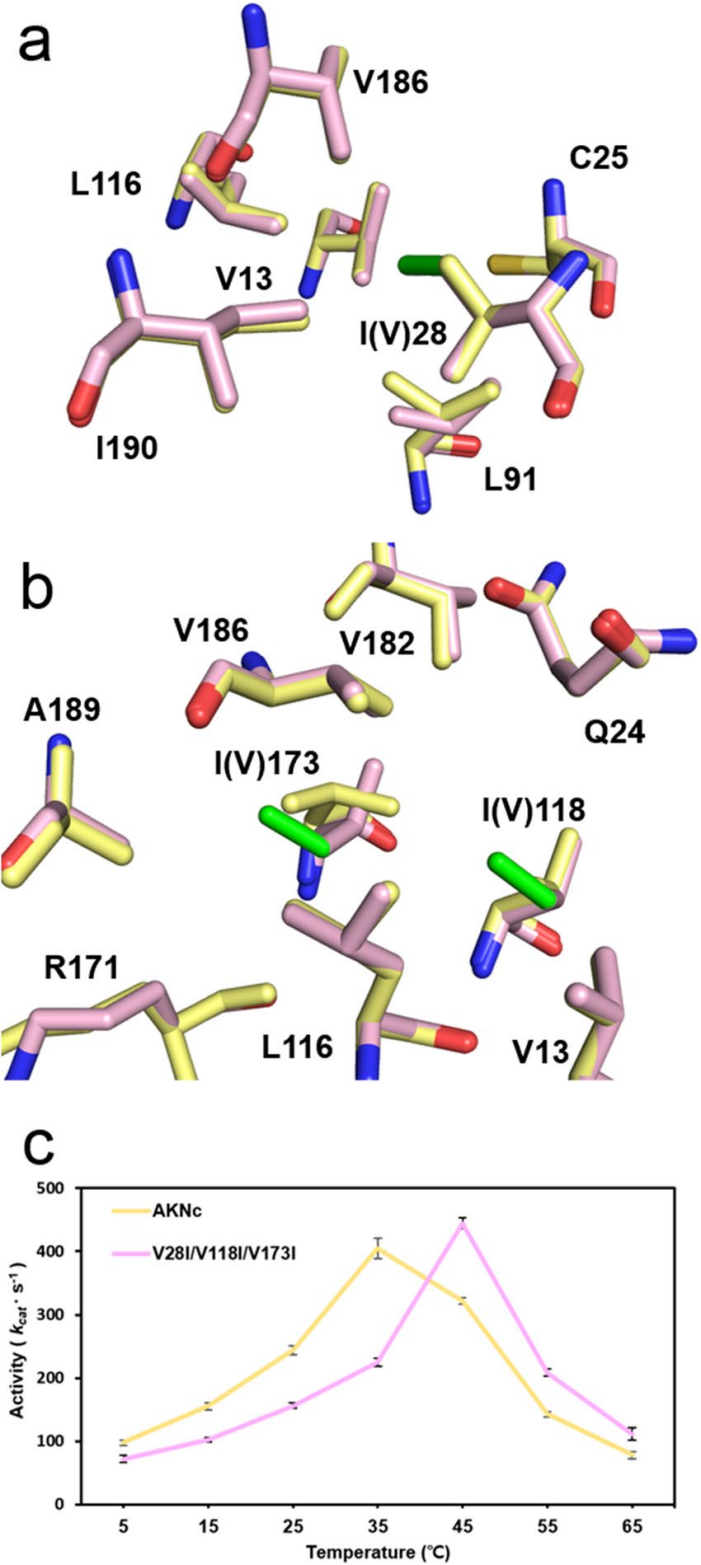
Optimization of hydrophobic CORE packing by Val-to-Ile substitutions in AKNc. (**a**,**b**) V28I (**a**) and V118I/V173I (**b**) substitutions improve CORE packing with other hydrophobic residues in the crystal structure of the V28I/V118I/V173I mutant of AKNc (pink). The three mutated Ile residues are highlighted in green. The structure of WT AKNc (yellow) is aligned with that of the mutant. (**c**) Temperature dependence of activity of WT AKNc and the V28I/V118I/V173I mutant. The activities in the direction of ATP formation were measured at various temperatures. The Val-to-Ile mutations reduced the catalytic activity at low temperatures (5–35 °C). At each temperature, three independent measurements were made. Data are represented as mean ± standard error of mean.

To confirm that thermal stabilization caused by the Val-to-Ile mutations resulted from the optimized hydrophobic CORE packing, we determined the crystal structure of a V28I/V118I/V173I mutant of AKNc (Fig. S3). Data collection and refinement statistics are listed in Table 2. The overall tertiary structure of the mutant was essentially identical to that of the WT. The RMSD value of Cα atoms between the WT AKNc and the mutant was 0.15 Å. In the mutant structure, the conformations of the residues neighboring the mutated Ile28 residue were almost indistinguishable from those around Val28 in the WT AKNc structure (Fig. 4a). This allows the added terminal methyl group (Cδ1) of the Ile28 side chain to interact hydrophobically with other residues in the CORE domain—such as Val13, Cys25, Leu91, Leu116, Val186, and Ile190—indicating the enhancement of the hydrophobic packing by the V28I mutation. The crystal structure of the AKNc mutant also revealed that the V118I and V173I mutations optimized the hydrophobic packing in the CORE of AKNc (Fig. 4b). The conformation of Ile118 was essentially identical to that of Val118 in the WT structure, with the exception of the extra methyl group (Cδ1) in its side chain, which makes additional hydrophobic contacts with Val13, Gln24, Leu116, and Val186 in β1, α1, β4, and α9, respectively, in the mutant structure. In contrast, the side chain conformation of the mutated Ile173 residue was distinct from that of Val173 in WT AKNc. The χ1 dihedral angle along the bond between the Cα and Cβ atoms of Ile173 was rotated ~120° relative to that of Val173 in the WT AKNc structure. The flipped side chain of Ile173 interacts hydrophobically with residues in β4, β5, and α9, including Leu116, Arg171, Val182, Val186, and Ala189. The hydrophobic contact between the two mutated residues (Ile118 and Ile173) is enhanced by the extension and conformational change of their side chains. The structural comparison of the WT and mutant AKNc revealed that the three Val-to-Ile mutations optimized the packing of the hydrophobic interior of the CORE domain.

Since our structural analyses are based on the Ap_5_A-bound structures, we also made T_m_ measurements of the WT and the triple mutant AKs in the presence of Ap_5_A (Table 3 and Fig. S1). The addition of Ap_5_A caused significant T_m_ increases for the AKs, indicating that the Ap_5_A binding stabilized the enzymes. Notably, the order in T_m_ was maintained for the WT and mutant enzymes regardless of the presence of Ap_5_A, but the T_m_ difference between them was reduced. For example, the V28I/V118I/V173I mutant of AKNc displayed higher T_m_ than the WT AKNc by 11.3 °C without Ap_5_A, whereas the mutant was more thermally stable than the WT enzyme only by 4.5 °C in the presence of Ap_5_A. In the previous studies of bacterial AK variants, the effects of T_m_ increase by applying multiple stabilization principles together were not strictly cumulative, and the magnitude of the T_m_ enhancement varied depending on the backgrounds to which the stabilizing factors were added^24,26,43^. Thus, the results from the T_m_ measurements with Ap_5_A seem to be consistent with the previous analyses, and, more importantly, confirm the validity of our structural analyses for the structural determinants of thermal stability.

**Table 3:** T_m_ values of fish AKs in the presence of Ap_5_A.

AK	Mutations	T_m_ (°C)	ΔT_m_ (°C)^a^
AKNc	WT	55.8	0.0
	V28I/V118I/V173I	60.3	4.5
AKDr	WT	61.8	6.0
	I28V/I118V/I173V	56.5	0.7

^a^Difference from the T_m_ of WT AKNc in the presence of Ap_5_A

### Optimization of hydrophobic CORE packing also affected catalytic function of AKNc

To examine the temperature dependence of the catalytic activity, we performed activity assays of WT AKNc and the mutant containing the three Val-to-Ile mutations (V28I/V118I/V173I) at various temperatures (Fig. 4c). The catalytic activity of the WT enzyme in terms of k_cat_ peaked at 35 °C and decreased afterwards, compared to 45 °C for the mutant AKNc, which was more catalytically active than the WT AKNc at high temperatures (45 °C and 55 °C). The increase of the activity at high temperature could be a consequence of the enhanced thermal stability of the mutant AKNc. The inactivation of the enzymes at high temperatures most likely resulted from thermal denaturation. However, the mutant showed considerably decreased activity at low temperatures (5–35 °C) compared to the WT enzyme, which cannot be explained by the difference in thermal stability between the WT and mutant AKs. It seems that the improvement in hydrophobic CORE packing by the Val-to-Ile mutations increased the structural stability at high temperatures and decreased the catalytic activity at low temperatures. The stabilizing mutations might make the enzyme too rigid, and the dynamic motion required for its catalytic function might be impeded at low temperatures due to the reduced flexibility.

The activity assays of AKDr and its I28V/I118V/I173V mutant also showed consistent results (Fig. S4). The AKDr mutant exhibited increased catalytic activities at low temperatures (5 °C and 35 °C) compared to the WT enzyme, and the temperature of maximum activity decreased (35 °C). However, the magnitude of the activity change at low temperatures by mutation was not as significant as in AKNc. This suggests that AKDr contains extra structural feature(s) maintaining the rigidity of its structure in addition to the hydrophobic interactions involving the Ile residues. In the crystal structure of AKDr, we identified a salt bridge connecting between Arg171 and Glu192 (Fig. S5), which is substituted to Ala192 in AKNc. Consistently, the T_m_ increase (11.3 °C) of AKNc by the three Val-to-Ile mutations was greater than the T_m_ decrease (8.9 °C) of AKDr by the reverse triple mutation (Table 1), supporting the role of the salt bridge. Taken together, our results suggest that activity and stability of fish AKs are inversely correlated, and disruption of hydrophobic packing may be a structural mechanism of cold adaptation as it could increase catalytic activity at low temperatures.

## Discussion

AKNc is useful for research on cold adaptation of psychrophilic enzymes for several reasons. The source organism of AKNc is the Antarctic fish *N*. *coriiceps*; therefore, the enzyme originated from a multicellular, eukaryotic psychrophile, whereas most previously characterized psychrophilic proteins were from psychrophilic microorganisms^4,5^. The conformational switching required for its enzymatic function also makes AKNc an attractive system to study the role of protein dynamics in cold adaptation as the maintenance of appropriate local and/or global motion is crucial for functioning at extreme temperatures. AK is a small protein that undergoes relatively large conformational changes upon substrate binding and product release (Fig. 1b), and has long been used as a model system for studying connections between structure, function, and dynamics^28–30,32,44–49^.

The ‘corresponding state’ hypothesis first proposed by Somero postulates that homologous proteins originated from organisms living at different environmental temperatures have comparable flexibilities and activities at their physiologically relevant temperatures^20,50^. Although this hypothesis has widely been accepted by the scientific community, whether the reduced stability of psychrophilic proteins is a consequence of maintaining the conformational flexibility necessary for functional activity at low temperatures, or a result of a lack of evolutionary pressure, remains unclear^51^. In previous studies of bacterial AKs, the AK variants generated exhibited both thermal stability at high temperatures and sufficient catalytic activity at low temperatures, suggesting that activity at low temperatures can be achieved without sacrificing stability, and thus the low stability of psychrophilic proteins is not an adaptive trait^46,52^. However, in this study, the stabilizing Val-to-Ile mutations in AKNc reduced the activity at low temperatures, indicating that stability and activity are coupled, and the decreased thermal stability of AKNc might be required for sufficient catalytic activity in cold environments.

This contradiction likely results from structural differences in the LID domain of AKNc and its bacterial homologues. In AKNc, the LID is a short loop of less than 10 residues (Figs 1a and 2a), whereas the bacterial AKs have LID domains of >30 residues that include several β-strands (Fig. S6). The long LID domain is crucial for the function of bacterial AKs, as LID opening was the rate-limiting step in catalysis^47^, and conformational heterogeneity within the LID domain was important in functional adaptation^53,54^. Hence, it is conceivable that stability and activity are governed independently by different domains of bacterial AKs with the large LID domains, but not in short isoforms such as AKNc. In a previous study, swapping of the CORE domains of AKs from mesophilic and thermophilic bacteria affected T_m_ values significantly, but did not affect the temperature dependence of activity, highlighting the spatial separation of stability and activity control in bacterial AKs^52^. Hence, for cold adaptation of bacterial AKs, residue substitutions in the LID domain are likely required to alter the LID dynamics, which are closely related to catalytic activity.

In the sequence alignment (Fig. 1a), the N- and C-terminal regions exhibited the greatest variability between AKNc and its homologues from tropical fishes. This observation suggests that these areas play more crucial roles in the temperature adaptation of AKs. The important role of the N- and C-terminal residues in overall thermal stability has already been noted for archaeal and bacterial AKs^23,24,44,55^. The chain folds of the AK homologues revealed that the first and last α helices (α1 and α9 in AKNc, respectively) in the amino acid sequence are located in close proximity^28,31,32^. Numerous polar and hydrophobic intramolecular interactions have been identified between the two terminal regions or involving residues from one of them^23,24,44^. In a previous study of archaeal AKs from the genus *Methanococcus*, chimeric AKs were constructed by exchanging the N- and C-terminal residues between mesophilic and hyperthermophilic homologues, and exhibited significant changes in T_m_ values (~20 °C) compared to the WT proteins^55^. Experimental evolution for thermal stabilization of a mesophilic bacterial AK by Shamoo and co-workers resulted in the generation of stable AK variants containing mutations at six different positions, five of which were located within the first or last 30 residues of the amino acid sequence^56^.

The Ile-to-Val mutations in the CORE domain of AKDr resulted in a significant decrease in thermal stability (Table 1). Several previous studies of the stabilization of the mesophilic *Bacillus subtilis* AK (AKBs) have reported the importance of hydrophobic CORE packing in thermal stability^26^. Comparative analyses with a thermophilic homologue enabled identification of a residue substitution (T179M) that enhanced hydrophobic interactions in the CORE domain^23^, which increased thermal stability when introduced to AKBs^26^. The experimental evolution of AKBs also generated several mutations (Q16L, T179I, and A193V)^56^ that stabilized the mesophilic target by improving the CORE packing^57^. Stable AKBs variants were previously designed based on a bioinformatic method of optimizing local structural entropy, an empirical descriptor of sequence heterogeneity^58^. The resulting AKBs mutants displayed increased apolar buried surface areas in the CORE domain, indicating the enhancement of hydrophobic contacts^43^. In a computational prediction followed by experimental validation, Wilson and co-workers tested 100 AKBs mutants and demonstrated that substantial thermal stabilization could be achieved by repacking of the hydrophobic CORE^59^.

In the present study, we discovered suboptimal hydrophobic packing in the CORE domain of the Antarctic fish AK. Comparative and mutational analyses demonstrated that imperfect hydrophobic CORE packing indeed results in reduced thermal stability and a shift in the temperature-activity profile. Our results suggest that modification of hydrophobic contacts is a key structural feature important for the cold adaptation of psychrophilic proteins, and may be used to engineer psychrophilicity in mesophilic enzymes.

## Methods

### Cloning, expression, and purification

Synthetic genes of the WT fish AK1 proteins were cloned into a pET28a vector with an N-terminal (His)_6_-maltose binding protein (MBP) tag and a tobacco etch virus (TEV) protease cleavage site. Mutant genes were generated by polymerase chain reaction (PCR) using mismatched primers. *Escherichia coli* BL21 (DE3) cells containing these constructs were cultured in LB medium at 37 °C until the optical density at 600 nm reached 0.7. Protein expression was then induced by the addition of 0.5 mM isopropyl-β-D-thiogalactopyranoside, followed by incubation at 17 °C for 16 h. The cells were harvested by centrifugation and resuspended in purification buffer (500 mM NaCl, 3 mM β-mercaptoethanol, 10% (w/v) glycerol, 20 mM Tris-HCl pH 7.0). After sonication and centrifugation, the supernatant was loaded onto a 5 mL HisTrap HP column (GE Healthcare, USA) equilibrated with purification buffer. The column was washed with purification buffer, and bound proteins were eluted by applying a linear gradient of imidazole (up to 500 mM). The (His)_6_-MBP tag was cleaved by TEV protease and separated using a HisTrap HP column. The proteins were further purified by size-exclusion chromatography using a HiLoad 16/60 Superdex 75 column (GE Healthcare, USA) equilibrated with size-exclusion chromatography buffer (300 mM NaCl, 3 mM dithiothreitol (DTT), 5% (w/v) glycerol, 50 mM HEPES pH 7.0).

### Measurement of T_m_ values

T_m_ values of AKs were determined by CD spectroscopy, as described previously^26^. CD traces at 220 nm were measured for 0.5 mg/mL AKs in 10 mM potassium phosphate pH 7.0 with or without 0.2 mM Ap_5_A. A Chirascan-plus CD Spectrometer (Applied Photophysics, UK) was used with a scanning rate of 1 °C/min. CD data were analyzed based on the protocol developed by John and Weeks^60^. Average values of three CD measurements at each temperature were differentiated to yield differential denaturation curves, which were fitted to parameters including T_m_ using a two-state transition model.

### Crystallization and structure determination

The WT and mutant AKNc proteins were crystallized under identical conditions. Their crystals were grown at 4 °C by the sitting-drop method from 18 mg/mL protein and 4 mM Ap_5_A in buffer (10 mM HEPES pH 7.0) mixed with an equal amount of reservoir solution (50% (v/v) polyethylene glycol 400, 200 mM lithium sulfate, 100 mM sodium acetate pH 4.5). The crystals were cryoprotected in the reservoir solution supplemented with 15% (v/v) ethylene glycol and flash-frozen in liquid nitrogen. The AKDr crystals were obtained at 20 °C by the sitting-drop method from 20 mg/mL protein and 4 mM Ap_5_A in buffer (10 mM HEPES pH 7.0) mixed with an equal amount of reservoir solution (2.5 M ammonium sulfate, 0.1 M sodium acetate pH 4.6). The crystals were cryoprotected in the reservoir solution supplemented with 20% (v/v) ethylene glycol and flash-frozen in liquid nitrogen.

Diffraction data from the AKNc and AKDr crystals were collected at 100 K at the beamlines 5 C and 7 A of the Pohang Accelerator Laboratory. Diffraction images were processed with HKL2000^61^. PHASER was used for molecular replacement phasing^62^. The structure of human AK1 (Protein Data Bank code 1Z83) was used as a starting model for the WT AKNc. Molecular replacement solutions for AKDr and the AKNc mutant were found with the WT AKNc structure. The final structures were completed using alternate cycles of manual fitting in COOT^63^ and refinement in REFMAC5^64^. The stereochemical quality of the final models was assessed using MolProbity^65^.

### Temperature-dependent activity assay

AK activity was measured at multiple temperatures in the direction of ATP formation as described previously, with minor modifications^52^. The enzymatic reaction was started by the addition of AK (1.1 ng/mL final concentration) to the reaction mixture (2.5 mM ADP, 1 mM glucose, 0.4 mM NADP^+^, 100 mM KCl, 2 mM MgCl_2_, 50 mM HEPES pH 7.4). After incubation at the indicated temperatures for 5 min, the reaction was stopped by the addition of 0.5 mM Ap_5_A. The amount of ATP produced by the reaction was determined by ATP-dependent NADP^+^ reduction to NADPH using hexokinase and glucose-6-phosphate dehydrogenase at room temperature. Average values of three independent measurements were reported with standard errors.

### Data availability

The atomic coordinates and structure factors were deposited in the Protein Data Bank^66^. The Protein Data Bank accession codes of AKNc, AKDr, and the V28I/V118I/V173I mutant of AKNc are 5X6 K, 5XZ2, and 5XRU, respectively.

## Electronic supplementary material

Supplementary Information
