# Supplementary material for: Structural analyses of adenylate kinases from Antarctic and tropical fishes for understanding cold adaptation of enzymes
Source: Sci Rep. 2017 Nov 22;7:16027. doi: 10.1038/s41598-017-16266-9 (PMC5700098; doi:10.1038/s41598-017-16266-9)
Supplement: Supplementary file 1 — Supplementary Information [file 41598_2017_16266_MOESM1_ESM.pdf]

Structural analyses of adenylate kinases from Antarctic and tropical fishes for understanding cold adaptation of enzymes

Sojin Moon, Junhyung Kim, and Euiyoung Bae

Supplementary Figures S1 to S6

Supplementary Figure S1

AKNc

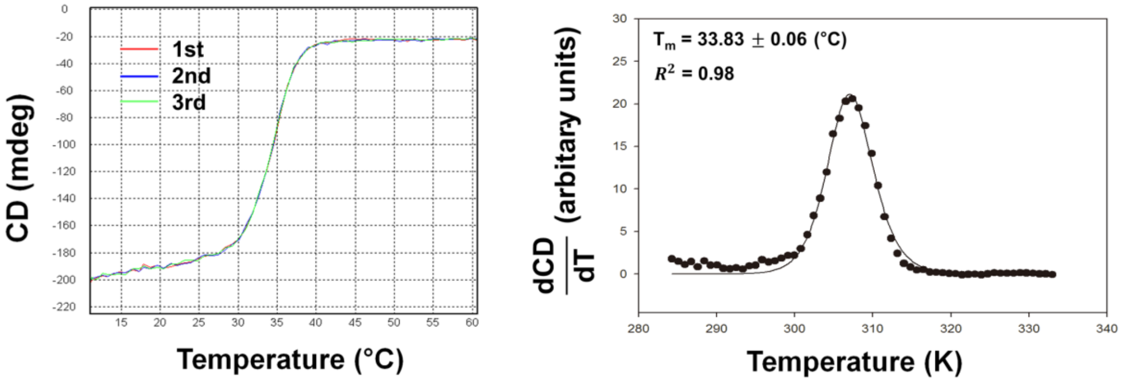

AKNc V28I

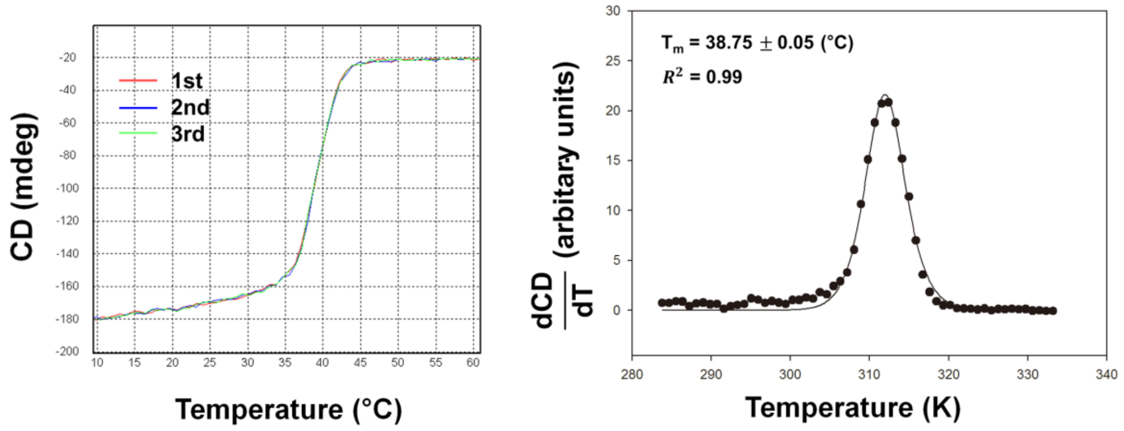

AKNc S48A

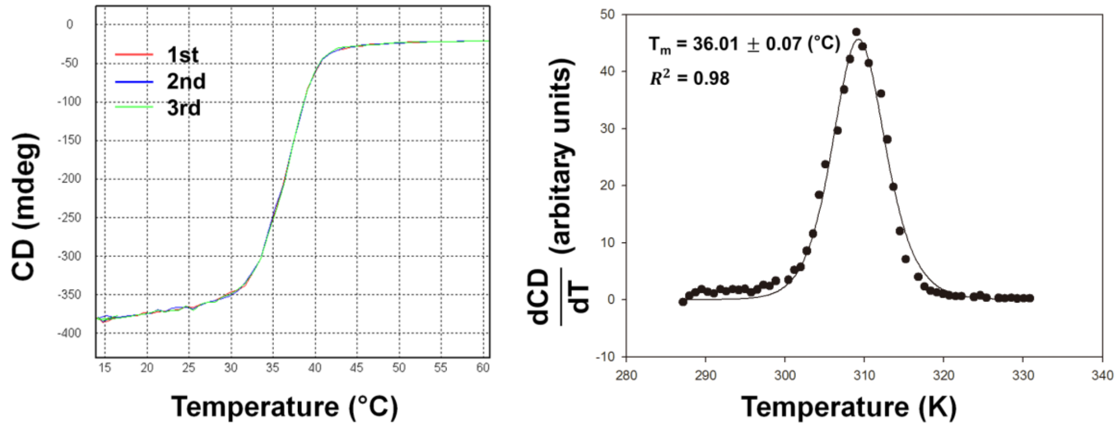

Supplementary Figure S1 (continued)

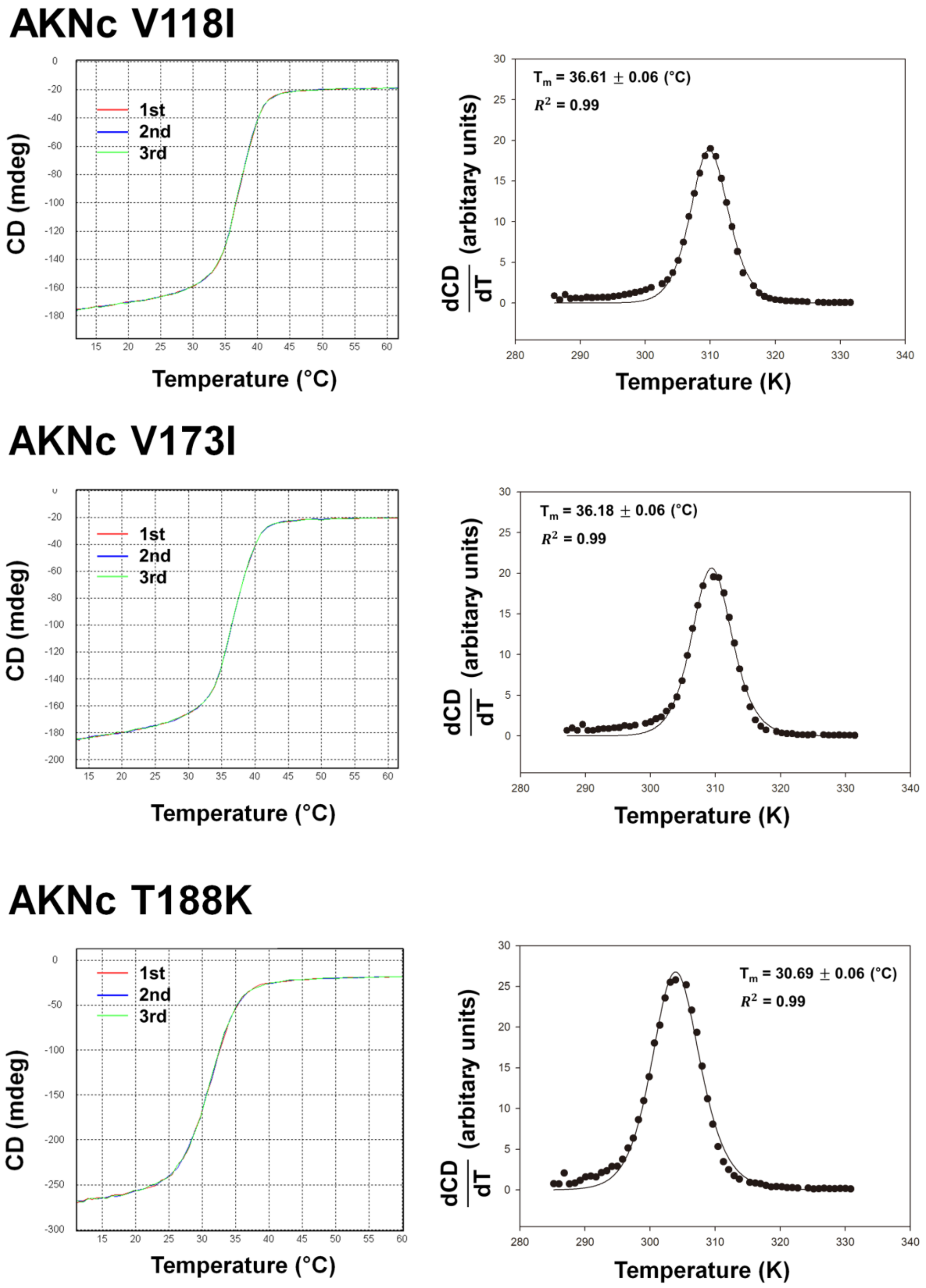

Supplementary Figure S1 (continued)

AKNc V118I/V173I

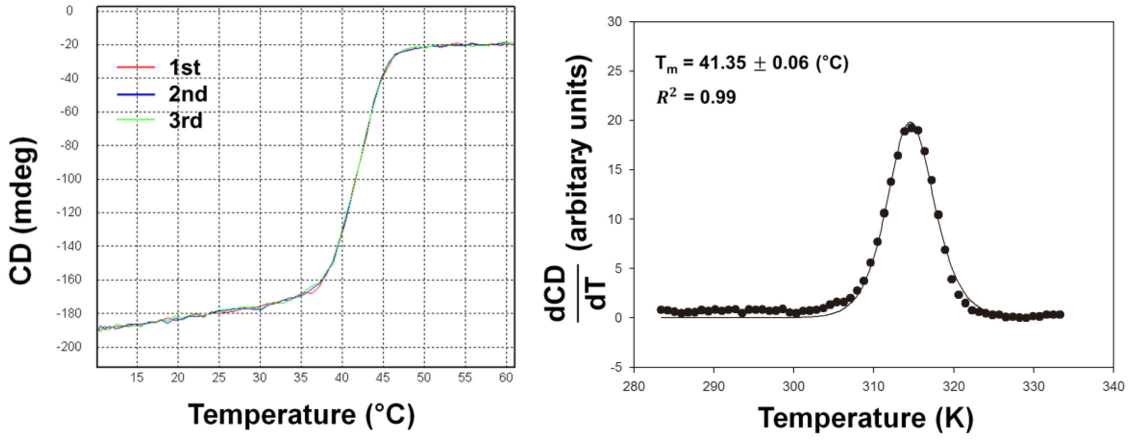

AKNc V28I/V118I/V173I

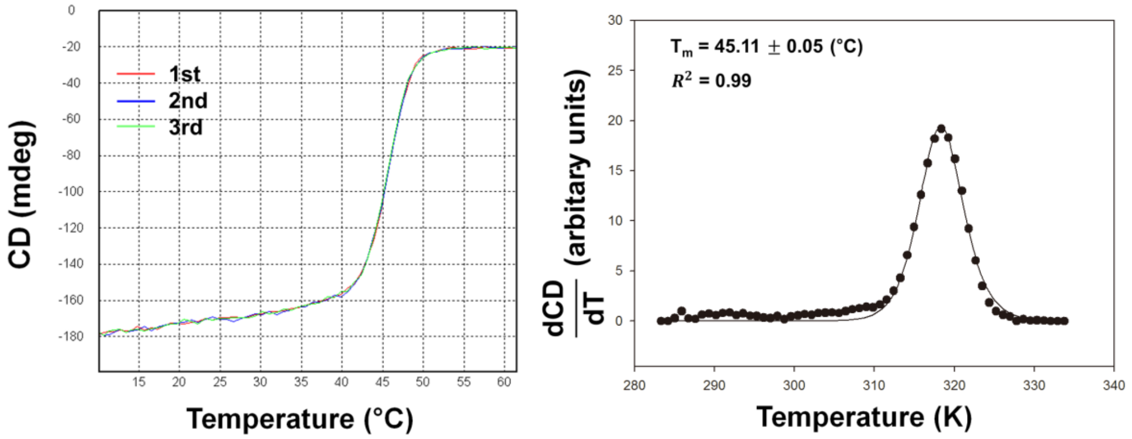

AKPr

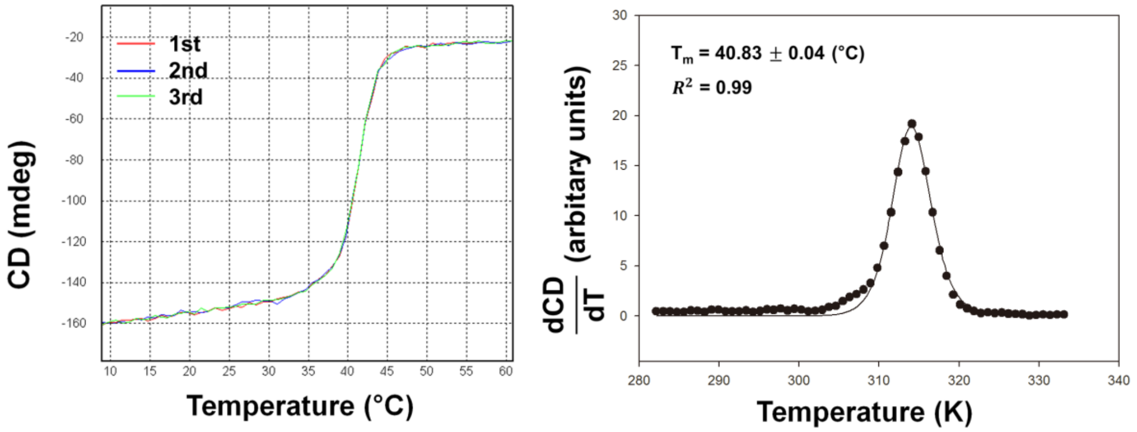

Supplementary Figure S1 (continued)

AKXm

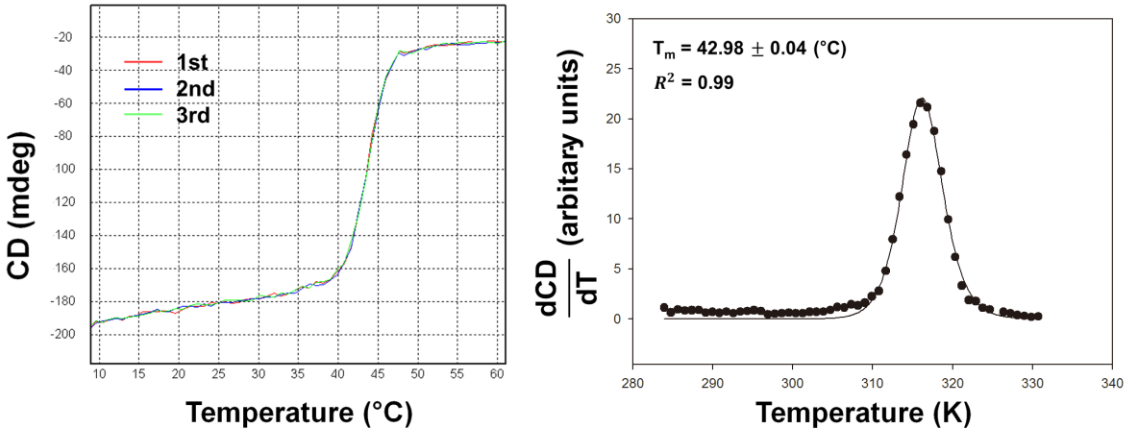

AKDr

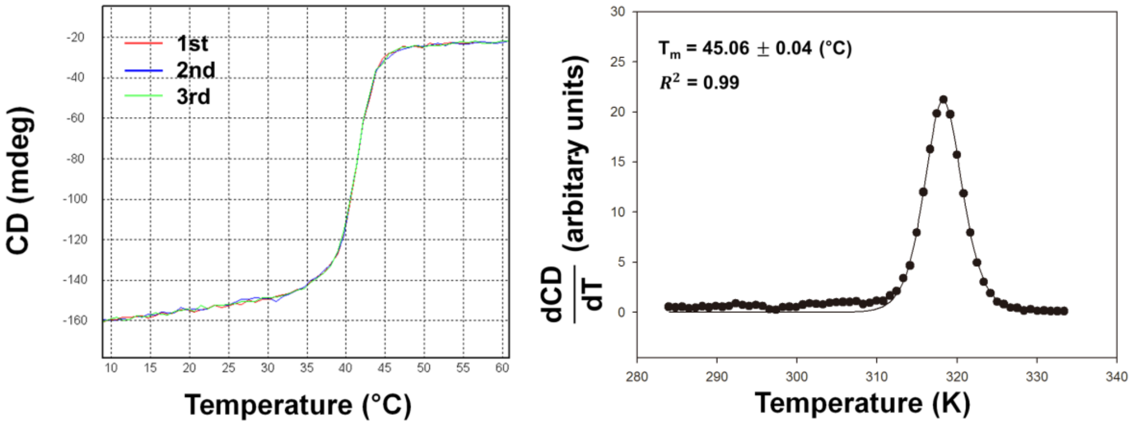

AKDr I28V/I118V/I173V

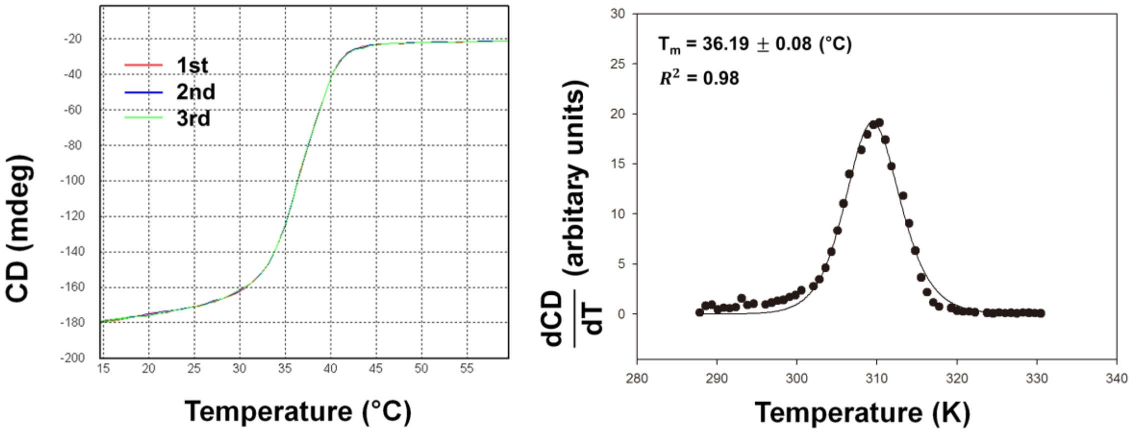

Supplementary Figure S1 (continued)

AKNc + Ap_5A

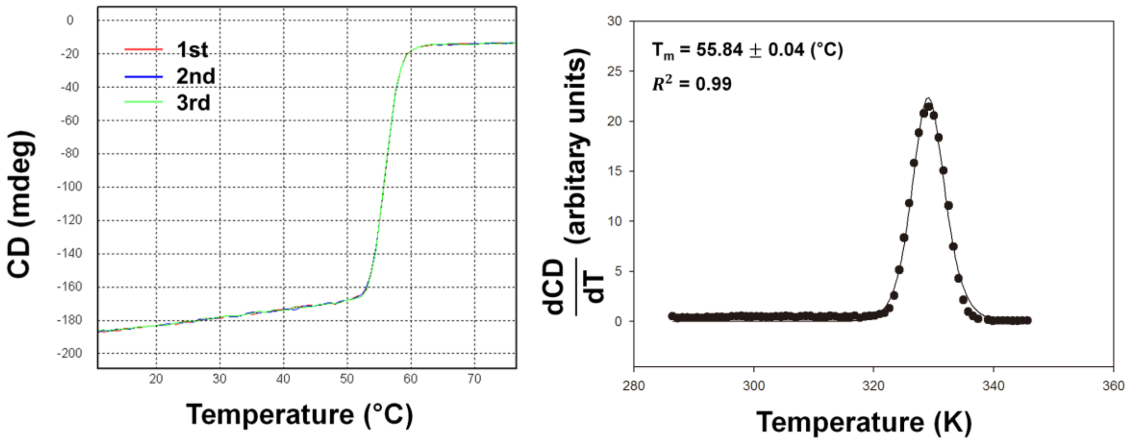

AKNc V28I/V118I/V173I + Ap_5A

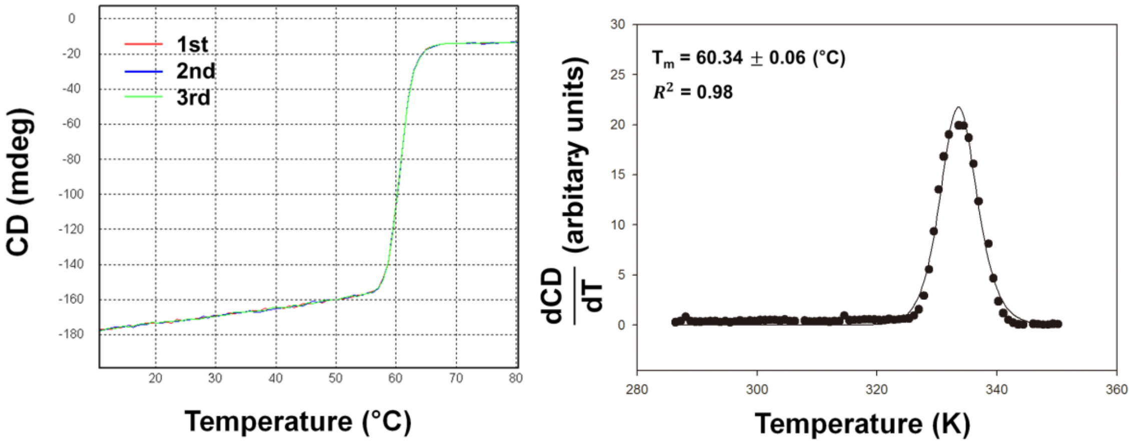

AKDr + Ap_5A

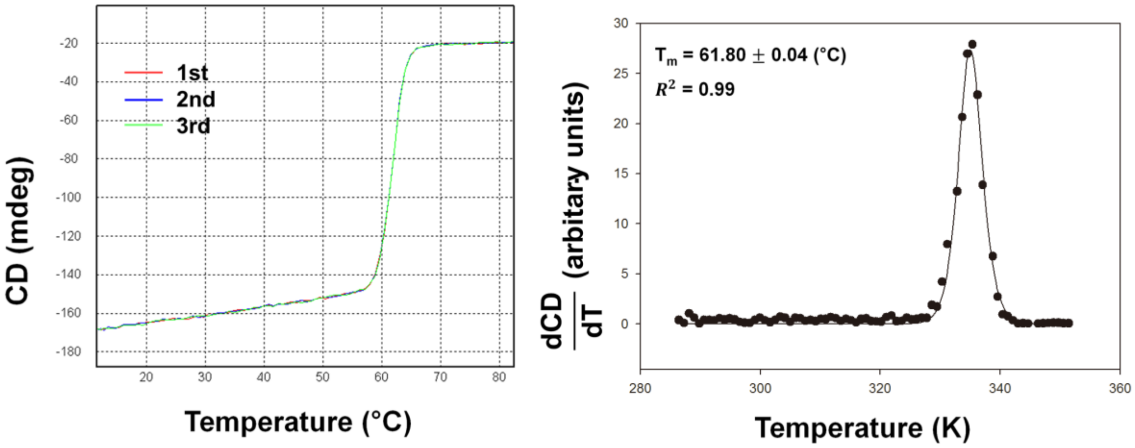

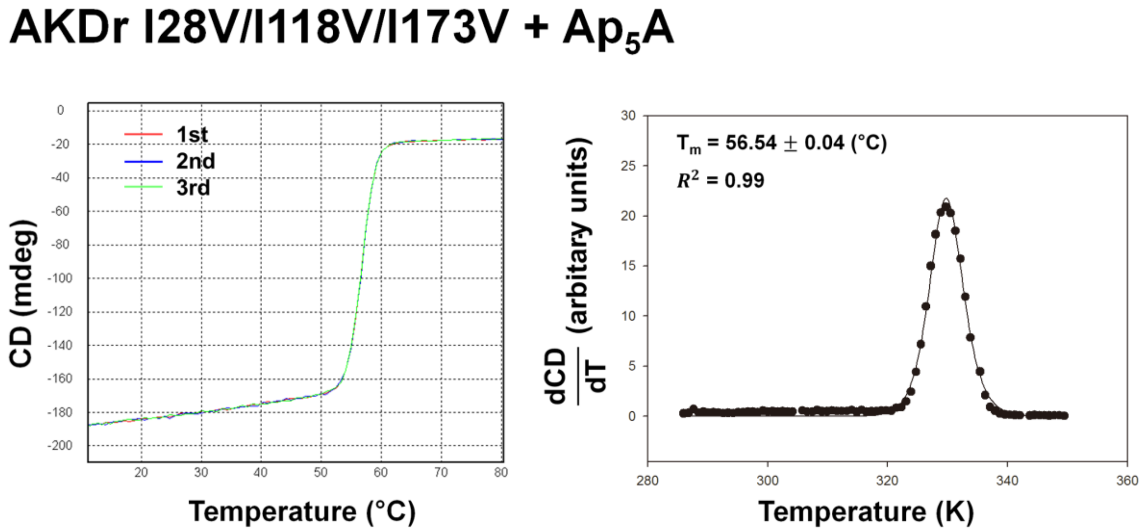

Figure S1. Thermal denaturation of AKs measured by CD spectroscopy. Original CD data (left) show three CD measurements of the samples at each temperature. Average values of the three measurements were differentiated to yield differential denaturation curves (right). Solid lines represent best fits to a two-state transition model for the differential denaturation curves. T_m's are indicated with their standard errors for the best-fit values in the non-linear regression. R^2 values of the regression are also indicated.

Supplementary Figure S2

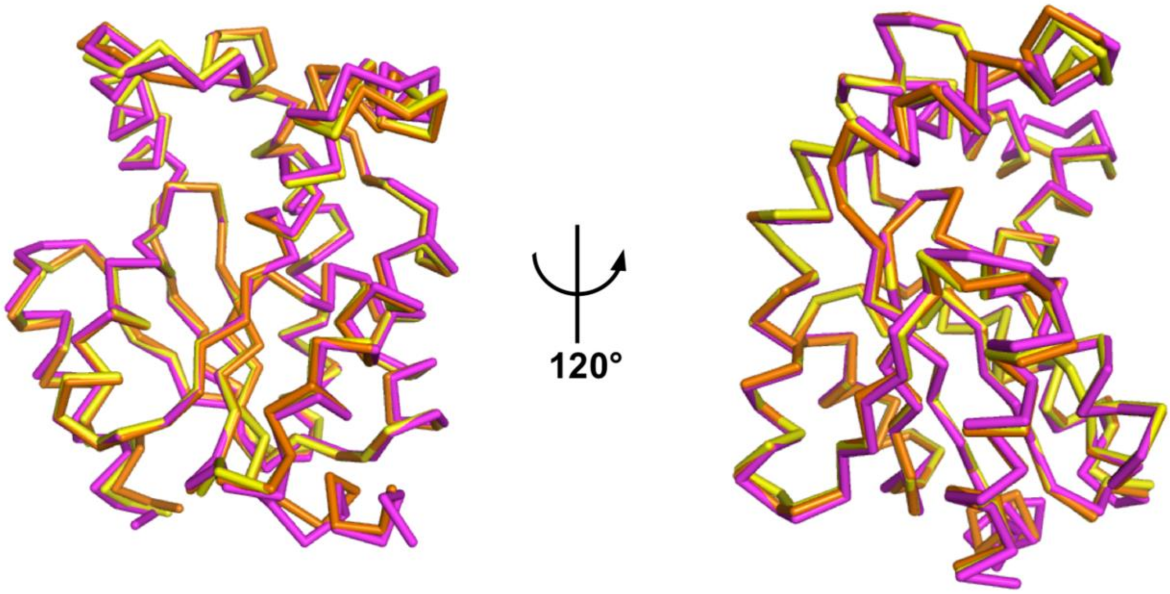

Figure S2. Structural alignment of AK1 homologues. AKNc, AKDr, and human AK1 (Protein Data Bank code 1Z83) are shown in yellow, orange, and magenta, respectively.

Supplementary Figure S3

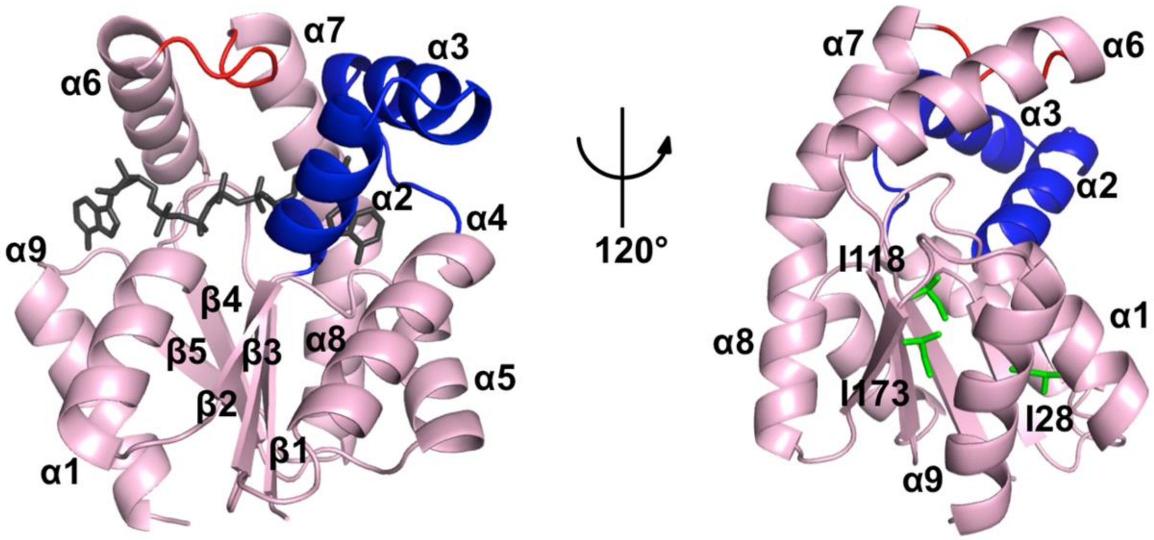

Figure S3. Overall structure of the V28I/V118I/V173I mutant of AKNc. The CORE (residues 1–38, 69–136, and 143–193), AMP_{bind} (residues 39–68), and LID (residues 137–142) domains are shown in pink, blue, and red, respectively. The bound Ap_5A molecules are shown in black (left). The mutated Ile residues are highlighted in green in stick representations (right).

Supplementary Figure S4

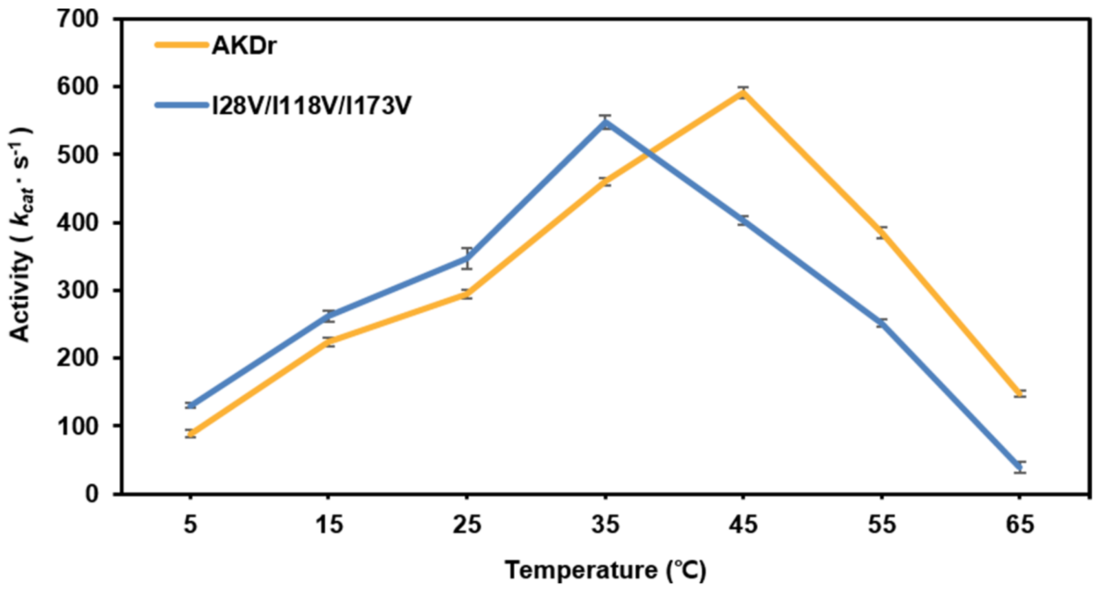

Figure S4. Temperature dependence of activity of WT AKDr and the I28V/I118V/I173V mutant. The activities in the direction of ATP formation were measured at various temperatures. At each temperature, three independent measurements were made. Data are represented as mean ± standard error of mean.

Supplementary Figure S5

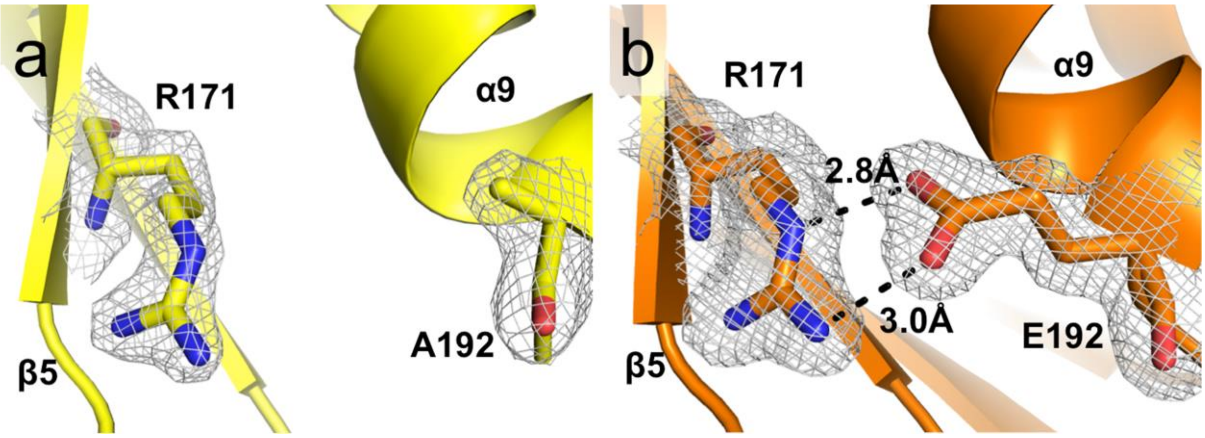

Figure S5. Interaction between residues 171 and 192 in AKNc (**a**) and AKDr (**b**). AKNc has Arg171 and Ala192. AKDr has Arg171 and Glu192, which form a salt bridge. The $2mF_{obs}$ - DF_{calc} map is contoured at 1.0 σ.

Supplementary Figure S6

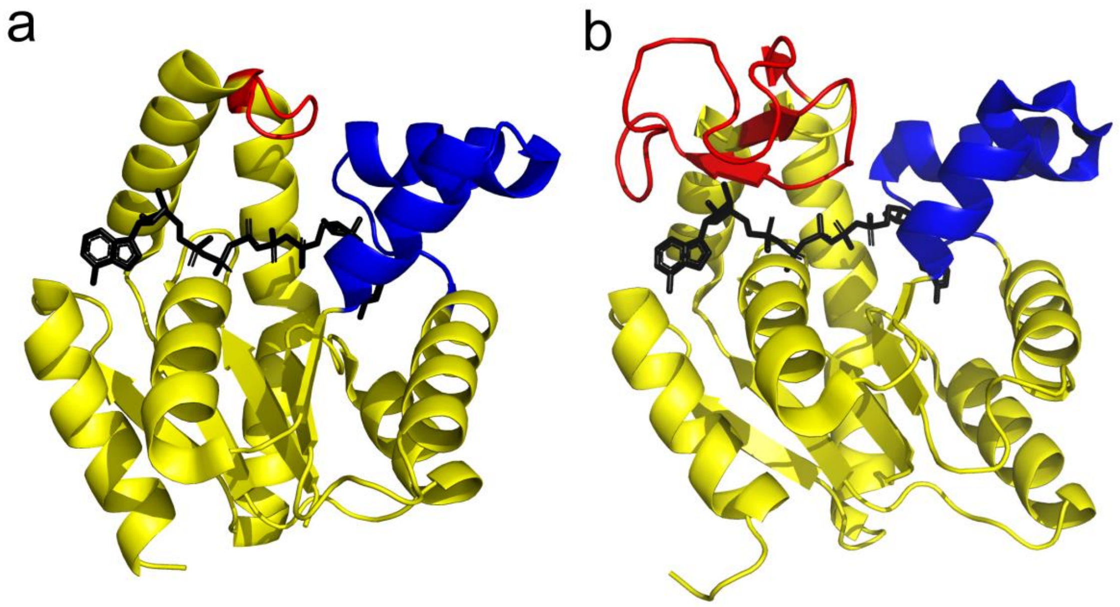

Figure S6. Structural comparison of AKNc and a bacterial AK with a long LID domain. Crystal structures of AKNc (**a**) and AK from a psychrophilic bacterium, *Bacillus globisporus* (**b**; Protein Data Bank code 1S3G). The CORE, AMP_{bind}, and LID domains are shown in yellow, blue, and red, respectively. Ap_5A molecules are shown in black.
